# Radio telemetry of butterflies: practical insights and critical tag-weight thresholds

**DOI:** 10.1186/s40462-025-00615-9

**Published:** 2025-12-09

**Authors:** Simon Heitzler, Sara Dallmöller, Heiko Hinneberg, Luis Ricardo Murillo-Hiller, Thomas K. Gottschalk

**Affiliations:** 1 University of Applied Forest Sciences Rottenburg, Schadenweilerhof, D-72108 Rottenburg a.N, Germany; 2 Faculty of Environment and Natural Resources, University of Freiburg, Tennenbacher Straße 4, D-79106 Freiburg, Germany; 3 Coordinación en Turismo Ecológico, SEDE de Guanacaste, Universidad de Costa Rica, Liberia, Costa Rica; 4 Centro de Investigación en Biodiversidad y Ecología Tropical (CIBET), Universidad de Costa Rica, San José, Costa Rica

**Keywords:** Butterfly telemetry, Flight performance, Insect tracking, Lepidoptera, Radio tag, Tag attachment, Tag weight

## Abstract

**Background:**

Radio telemetry offers new opportunities for studying the movement of insects. One important prerequisite for using radio tags to study butterfly movement ecology is that tag weight and attachment do not significantly affect butterfly flight performance. Despite recent applications of telemetry in butterflies, a systematic evaluation of tag-to-body-weight thresholds for successful tagging has been lacking.

**Methods:**

We tested ultra-light radio tags (0.13 g) on 117 individuals of 18 butterfly species under greenhouse and field conditions. Tag-to-body-weight ratios ranged from 5.6% to 77.8%. We used generalized linear mixed-effects models to identify predictors of flight success and used ROC analysis to determine the critical tag-to-body-weight threshold. Tag retention was also compared between thoracic and abdominal attachment sites.

**Results:**

We found that a threshold of approximately 20% of body weight marks a critical point beyond which flight performance declines significantly. Abdominal tag attachment proved more reliable and stable than thoracic attachment, with lower detachment rates.

**Conclusions:**

This study presents the first comprehensive evaluation of tag-to-body-weight thresholds and attachment methods in butterfly telemetry. The results provide practical guidance for planning radio telemetry studies of butterflies and for conducting further methodological research, such as into the effects of tagging on butterfly behavior, body condition, survival, and reproduction.

**Supplementary Information:**

The online version contains supplementary material available at 10.1186/s40462-025-00615-9.

## Background

The movement patterns and habitat use of butterflies (Lepidoptera: Rhopalocera) are key aspects of their ecology and conservation. However, they have so far been insufficiently studied for many species due to the species’ elusive nature and high mobility and the scientists’ limitations in methodology. To date, most evidence of butterfly dispersal results from extensive capture-mark-recapture (CMR) studies [1–6]. These studies have provided valuable insights into butterfly ecology, including dispersal potential and population dynamics. However, information about individual movement from CMR studies typically relies on random recaptures of those individuals that remain within a predefined study area. In addition, individuals can only be detected with a high probability in a CMR study when they are active. This limits our understanding of complex movement patterns and the factors that influence butterfly habitat use.

Radio telemetry is a promising tool to overcome these limitations of CMR studies, as it enables continuous tracking of individuals over longer time periods. In radio telemetry studies animals are equipped with tags that transmit radio waves, which can then be recorded by receivers [7]. This technique allows tracking the movement of individual animals over time, providing detailed data on movement patterns, habitat use, and resource utilization [7]. Originally developed for larger animals like birds and mammals, radio telemetry has recently become feasible for smaller organisms such as arthropods, including butterflies, due to advances in technology and the development of lightweight tags [8, 9].

Several studies have investigated the use of radio telemetry in insects, including crickets [10], beetles [11], bees [12, 13], moths [14, 15], and dragonflies [16, 17]. Batsleer et al. and Kissling et al. summarized the current state-of-the-art on arthropod tracking [9, 18]. Despite butterflies being among the most studied insects (e.g [19, 20]), comparatively few studies have employed telemetry for this group. To our knowledge, telemetry studies of butterflies have primarily focused on the Monarch butterfly (*Danaus plexippus*) and the Golden Birdwing (*Troides aeacus*) [17, 21, 22]. The Monarch butterfly is a migratory species and a well-studied insect, often used as an example of insect migration behavior [23].

For a successful radio telemetry study, it is crucial to ensure that the tags used do not or only minimally interfere with individual movement. A key factor in this regard is the weight of the tag [9]. For vertebrates, the general rule is that the tag should not exceed 5% of the individual’s body weight [24]. Such a rule of thumb does not exist for insects, as a systematic study of their transport capacities has not yet been carried out. However, some insects, particularly beetles, wasps and bees, exhibit remarkable carrying capacities compared to vertebrates [25, 26].

In their review, Batsleer et al. found that most of the 173 studies they reviewed did not quantify the potential impacts of tags on terrestrial arthropods; in 40% of the studies, potential effects were not addressed [18]. This is an important shortcoming, because the few studies that investigated the impacts of tags on insects in detail found that tags can significantly compromise insect movement. Boiteau and Colpitts concluded that tags exceeding 23–33% of body weight impair upward flight in Colorado potato beetles (*Leptinotarsa decemlineata*) [27]. Nonetheless, beetles exhibit comparatively lower flight efficiency than other insects, potentially due to the weight of their sclerotized elytra [25]. Batsleer et al. found that tags weighing 27.5% of body weight reduced flight activity in *Bembix rostrata*, especially in individuals with higher wing loading [18].

Fisher et al. found no significant differences between tagged (0.22 g tag weight) and untagged butterflies, but tagged individuals appeared to spend less time flying [21]. Liégeois et al. reported that tags weighing 8–13% of body weight did not impede flight in the moth species *Paysandisia archon*, although they defined no specific criteria for flight impairment [14]. Wang et al. did not directly test the impact of the tag on butterfly flight behavior [22]. However, they referenced other studies that have successfully used lightweight tags on various insects [12, 14, 28], indicating minimal interference with natural behaviors. They noted that tags weighing 0.2 g (approximately 20% of the average body weight of their study species) have been used in studies involving bumblebees, beetles, dragonflies, crickets, and moths, suggesting that the weight of the tag used in their study is unlikely to significantly affect the butterflies’ flight patterns ([12] (bumblebees); [9] and [14] (moths); [28](butterflies)). Knight et al. highlighted that radio tags with up to 49% of the insects’ body weight may hinder the natural flight of *D. plexippus* [17]. In particular, individuals were unable to sustain flight immediately after release. Menz et al. studied the hawkmoth *Acherontia atropos* and found no flight impairment with tags weighing 8–15% of body weight [15]. Srygley and Kingsolver investigated the influence of an additional weight (15% of body weight) on the flight performance of the butterfly *Anartia fatima* [28]. The added weight, in the form of a tin alloy, had no consistent impact on flight speed, predator escape ability, or overall survival rate of the butterfly species. In addition, Kingsolver and Srygley conducted experiments on two butterfly species, *Pontia occidentalis* and *Colias philodice*, in which extra weights corresponding to 10–16% of their body weight were added [29]. In both species, the likelihood of initiating flight decreased with the added weight, indicating reduced flight activity.

Although these studies give important hints on the potential impact of tags on butterfly behavior, many open questions remain, e.g. what is the effect of tag attachment and where is the best position on the butterfly body to attach a tag? Is there a threshold tag-to-body weight ratio that still allows virtually unaffected movement?

We have addressed these blind spots of previous butterfly telemetry studies. The main objective of this study is to define an operational tag-to-weight ratio for the use of radio telemetry in butterflies. We selected 18 butterfly species native to Central Europe (Germany) and Central America (Costa Rica) for our study. We combine flight tests in a greenhouse and telemetry studies in the field to gain insights into the practicability and requirements of radio telemetry of butterflies. We tested how increasing tag-to-body-weight ratio affects the flight performance of butterflies, particularly their ability to maintain stable and prolonged flights, with the aim of identifying critical thresholds for the use of telemetry in butterflies. Additionally, we tested different tag attachment sites to assess both tag retention and potential harm to the butterflies.

## Methods

### Study subjects

This study included 18 different butterfly species, from the families Nymphalidae and Papilionidae (Table 1). The species were selected based on their body weights to cover a broad gradient of tag-to-body weight ratio.

**Table 1:** Overview of the 18 butterfly species studied with family, capture location, number of individuals studied, and average body weight (g)

Species	Family	Capture location	*N*	Average body weight (g)
*Archaeoprepona demophon*	Nymphalidae	Germany, Potsdam Biosphere (greenhouse)	1	0.78
*Archaeoprepona demophoon*	Nymphalidae	Costa Rica, El Zota	3	1.46
*Argynnis paphia*	Nymphalidae	Germany, Rottenburg	2	0.18
*Brintesia circe*	Nymphalidae	Germany, Tübingen	7	0.26
*Caligo memnon*	Nymphalidae	Germany, Biosphäre Potsdam (greenhouse)	30	1.24
*Caligo oedipus*	Nymphalidae	Costa Rica, El Zota	4	1.01
*Historis odius*	Nymphalidae	Costa Rica, El Zota	2	1.31
*Hypolimnas monteironis*	Nymphalidae	Germany, Biosphäre Potsdam (greenhouse)	1	0.45
*Idea leuconoe*	Nymphalidae	Germany, Biosphäre Potsdam (greenhouse)	1	0.56
*Iphiclides podalirius*	Papilionidae	Germany, Zossen	2	0.30
*Kallima inachus*	Nymphalidae	Germany, Biosphäre Potsdam (greenhouse)	1	0.48
*Morpho helenor*	Nymphalidae	Germany, Biosphäre Potsdam (greenhouse); Costa Rica (greenhouse); Costa Rica, El Zota	45	0.65
*Nymphalis antiopa*	Nymphalidae	Germany, Rottenburg	2	0.34
*Papilio lowii*	Papilionidae	Germany, Biosphäre Potsdam (greenhouse)	1	0.64
*Papilio machaon*	Papilionidae	Germany, Rottenburg	8	0.29
*Papilio memnon*	Papilionidae	Germany, Biosphäre Potsdam (greenhouse)	1	0.55
*Parthenos sylvia*	Nymphalidae	Germany, Biosphäre Potsdam (greenhouse)	5	0.38
*Vanessa atalanta*	Nymphalidae	Germany, Zossen	1	0.32

### Butterfly sampling

Butterflies were mainly captured using hand nets. In Costa Rica, baited traps containing fermenting fruits were also used [30]. Traps were frequently monitored to minimize stress.

### Environmental conditions

Butterflies were captured during warm, sunny conditions at times of high butterfly activity. In Germany, butterflies were captured between 10:00 a.m. and 5:00 p.m., in Costa Rica between 5:30 a.m. to 2:00 p.m. and, additionally, from 5:00 p.m. and 6:30 p.m. to account for the specific circadian rhythm of *Caligo* butterflies, which are flying at dawn and dusk [31]. Study sites were the Biological Station El Zota in Costa Rica (10.557269° N, 83.736178° W), three areas in Germany (near Tübingen, 48.506172° N, 8.982459° E; near Rottenburg, 48.453174° N, 8.989955° E; Zossen, 52.216311° N, 13.476030° E) and in the greenhouse Potsdam Biosphere (52.418359° N, 13.049838° E). At the greenhouse captures were conducted under 26–28 °C with 75–85% humidity.

### Tag specifications and radio telemetry technique

We used ultra-lightweight radio tags (NanoPin Feather, 0.13 g, Lotek Wireless Inc.), capable of transmitting on frequencies between 150.300 and 150.600 MHz. To our knowledge, this is the lightest commercial radio tag currently available. The tags have a minimum battery life span of seven days. Signals were tracked using the Biotracker VHF Receiver (Lotek Wireless Inc.) and a Yagi antenna (LITEFLEX 3-Element VHF Yagi Antenna, Lotek Wireless Inc.). The tags had a signal range of approximately 400–500 m in dense rainforest and up to 1 km in open terrain from elevated positions. However, signal precision was affected by obstacles such as buildings, power lines, and electric fences, which sometimes introduced interference.

### Weight measurements

Body weight of butterflies was recorded to two decimal places with a portable scale (Kern PCD 250-3). Individuals were immobilized in an envelope during weighing. All individuals involved in this study were weighed, except for the individuals of *Brintesia circe* and *Papilio machaon*. For *B. circe*, one individual was directly weighed, while the species mean weight was assigned to the other individuals. These mean values result from weight measurements of 12 (*B. circe*) and nine (*P. machaon)* conspecifics, which were not used for tagging.

### Tag attachment

Tags were attached using two types of adhesives: cyanoacrylate glue (Uhu blitzschnell super glue) and UV-curable glue (Bondic UV-light curing plastic). Cyanoacrylate glue required more time to dry but offered a longer-lasting bond, while UV glue facilitated faster handling. In the greenhouse, UV glue was exclusively used to enable tag removal without harming the butterflies. The thorax or abdomen were carefully freed of scales by applying a small amount of adhesive, which caused the scales to stick to it, before being removed. Handling times varied depending on the specific procedures performed. The tag attachment took between 1:00 and 4:30 min (mean 2:50).

### Tag attachment sites

**Fig. 1:**
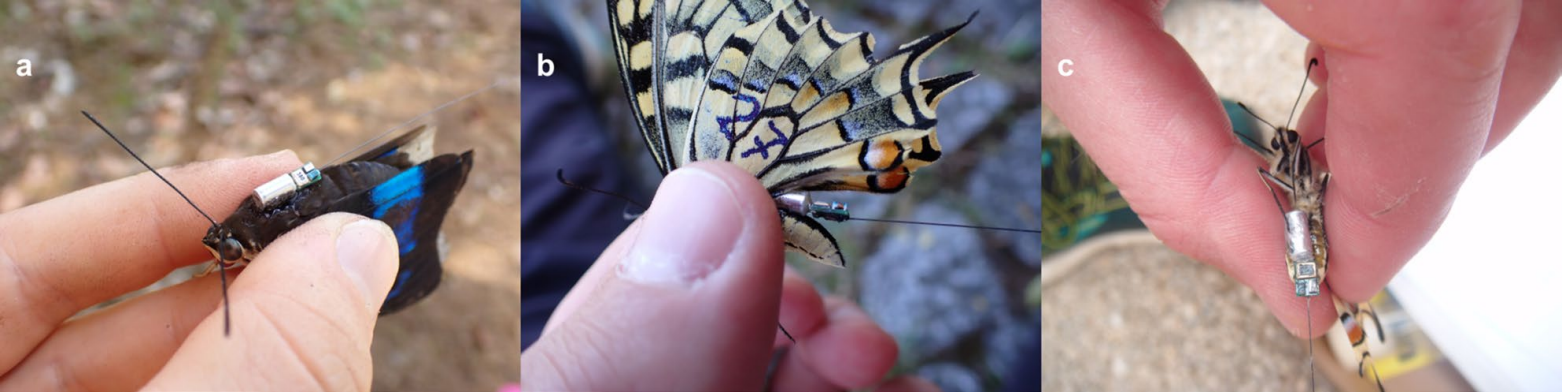
Tag attachment: (**a**) dorsal thorax attachment (*Archaeoprepona demophoon*), (**b**) dorsal abdomen attachment (*Papilio machaon*), and (**c**) ventral abdomen attachment (*P. machaon*)

Tags were attached to three different locations: the dorsal thorax, the dorsal abdomen, and the ventral abdomen (Fig. 1). The effect of attachment site was then assessed in terms of stability, ease of attachment, and flight behavior.

### Field tracking

Field tracking was conducted on foot, by bicycle or by car, depending on the terrain and butterfly behavior. On-foot tracking was conducted in both Germany and Costa Rica to allow close observation, bicycles were used in Tübingen and Rottenburg, Germany, for *P. machaon* covering longer distances. In Zossen, Germany, a car was used for *Iphiclides podalirius* able to travel quickly over greater distances. This approach was conducted to maintain visual contact with butterflies to study behavior, resource and habitat use. If the signal was lost, an attempt was made to relocate the transmitter from an elevated point. If this succeeded, triangulation was used to determine the butterfly’s exact location.

### Data collection

Geographic locations were recorded using a Garmin GPSMAP 64s device. In the field, the first few minutes of flight were observed by at least two people in order to be able to determine the direction of movement in case of a quick escape from the place of release.

### Evaluation of the initial flight performance

The initial flight phase was assessed for all butterflies, both in the greenhouse and in the field. Flight performance of the initial flight phase was categorized using two distinct levels:

1. Impaired initial flight: The butterfly exhibits any impairment during take-off or initial flight, including slow lift-off, unstable flight, short flight distances, or reduced control.
2. Strong initial flight: The butterfly showed a strong takeoff behavior with immediate and stable flight, demonstrating no noticeable interference from the tag.

These initial flight phase categories provided insight into the immediate impact of the tag attachment on flight behavior under both controlled and natural conditions.

### Field tracking and extended flight evaluation

The extended flight evaluation was conducted only for ten species captured in the field, as flight distances were strongly limited in the greenhouse. In the field, we evaluated longer-term flight performance over a period of up to six days. Extended tracking of selected species provided insights into the potential long-term effects of tags. This approach enabled a comprehensive understanding of both immediate and long-term impacts of tagging on flight behavior.

Flight performance was assessed using five levels, considering both flight distance and observed behaviors during the entire tracking period:

1. Attempted flight: The butterfly briefly lifted off but could not sustain flight, returning to the ground. It showed significant impairment, being unable to perform coordinated or sustained flights.
2. Struggling flight: The butterfly managed short distances but struggled to gain altitude, indicating that the tag noticeably affected flight.
3. Impaired but capable flight: The butterfly was capable of sustained flight over longer distances but exhibited extended resting periods and slower takeoffs, suggesting tag influence.
4. Weighted flight: The butterfly displayed generally normal flight behavior but with limited gliding. The tag altered flight dynamics, leading to reduced speed and changes in flight angle.
5. Normal flight: The butterfly exhibited no noticeable impairment, with normal agility and flight speed, similar to untagged individuals.

For extended field observations, flight distances and durations were documented to assess overall flight performance (Table 2).

**Table 2:** Species, individual ID, observed flight distances (m), and observation durations (days) during extended field observations. For some individuals, flight performance was observed but the exact flight distance could not be measured due to field constraints. These cases are indicated by “-” in the observed flight distance column

Species	Individual ID	Observed flight distance (m)	Observation time (days)
*Archaeoprepona demophoon*	f1	-	1
*Archaeoprepona demophoon*	f2	288	5
*Archaeoprepona demophoon*	f3	706	4
*Argynnis paphia*	f4	0	1
*Argynnis paphia*	f5	0	1
*Brintesia circe*	f10	-	1
*Brintesia circe*	f11	283	3
*Brintesia circe*	f12	362	5
*Brintesia circe*	f6	215	1
*Brintesia circe*	f7	-	1
*Brintesia circe*	f8	-	1
*Brintesia circe*	f9	-	3
*Caligo oedipus*	f13	143	3
*Caligo oedipus*	f14	529	6
*Caligo oedipus*	f15	50	1
*Caligo oedipus*	f16	20	2
*Historis odius*	f17	291	3
*Historis odius*	f18	70	1
*Iphiclides podalirius*	f34	223	3
*Iphiclides podalirius*	f35	3419	6
*Morpho helenor*	f19	-	2
*Morpho helenor*	f20	-	2
*Morpho helenor*	f21	75	2
*Morpho helenor*	f22	-	1
*Morpho helenor*	f23	-	2
*Nymphalis antiopa*	f24	300	4
*Nymphalis antiopa*	f25	233	5
*Papilio machaon*	f26	207	1
*Papilio machaon*	f27	299	1
*Papilio machaon*	f28	2543	3
*Papilio machaon*	f29	864	2
*Papilio machaon*	f30	171	3
*Papilio machaon*	f31	9378	3
*Papilio machaon*	f32	1139	2
*Papilio machaon*	f33	1000	1
*Vanessa atalanta*	f36	-	1

**Fig. 2:**
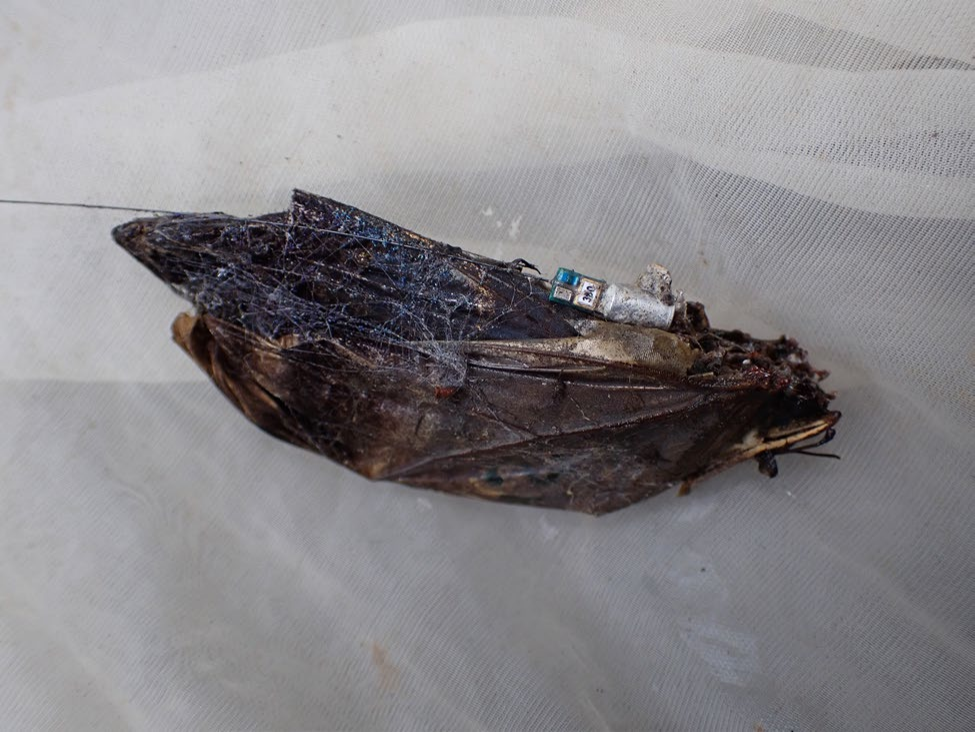
Dead *Caligo oedipus* female with tag attached found in a spider web

Observations of the tagged butterfly were conducted until the signal was lost, the tag fell off, or the butterfly was found dead (Fig. 2).

### Data analysis

Spatial data were stored, managed, and analyzed using QGIS (Version 3.34.5). Flight distances were calculated by connecting sequential GPS locations into lines and measuring their length using the Field Calculator.

All statistical analyses were conducted in R version 2023.03.0 [32]. We used the packages lme4 [33] for model fitting, pROC [34] for ROC analysis, and DHARMa [35] for simulation-based residual diagnostics.

We modelled initial flight capacity (binary outcome: 1 = strong flight, 0 = impaired flight) using a generalized linear mixed-effects model (GLMM) with binomial error distribution and a logit link function. The main predictor was the tag-to-body-weight ratio (tag weight as a percentage of body weight). Species was included as a random intercept to account for potential differences in baseline flight performance among taxa. Key model assumptions were evaluated prior to model fitting. Linearity of the logit was assessed using the Box-Tidwell method and visual inspection of residual plots. Although the test indicated a moderate deviation from strict linearity, the untransformed linear term was retained based on model parsimony and empirical justification from the diagnostic plots. The outcome variable was binary, and no perfect separation was detected across the predictor. The model was fitted using maximum likelihood estimation (Laplace approximation). Residual diagnostics were performed using simulated residuals generated with the DHARMa package, including tests for dispersion and uniformity as well as graphical assessment of model fit. In addition, the distribution of random intercepts was visually inspected for approximate normality.

To assess predictive performance, we generated a predicted probability curve across the observed range of the tag-to-body-weight ratio. A receiver operating characteristic (ROC) curve was constructed using the pROC package, and the optimal probability threshold was determined based on the Youden index. This value was then back-transformed to obtain the corresponding cut-off point in tag-to-body-weight ratio.

To test model robustness, we fitted an alternative model with tag-to-body-weight ratio as a fixed effect and a random slope by species and an observation-level random effect (OLRE) to account for potential overdispersion. Model fit was compared using Akaike’s Information Criterion (AIC) and the Bayesian Information Criterion (BIC), respectively.

Extended flight capacity was analyzed with a cumulative link mixed model (CLMM, package ordinal) using tag-to-body-weight ratio as a fixed effect and species as a random effect. Fisher’s Exact Test was used to assess whether tag detachment rates were associated with the attachment site (dorsal thorax, dorsal abdomen, ventral abdomen). Detachment rate was defined as the proportion of individuals for which the tag detached during the observation period.

## Results

### Initial flight performance

**Fig. 3:**
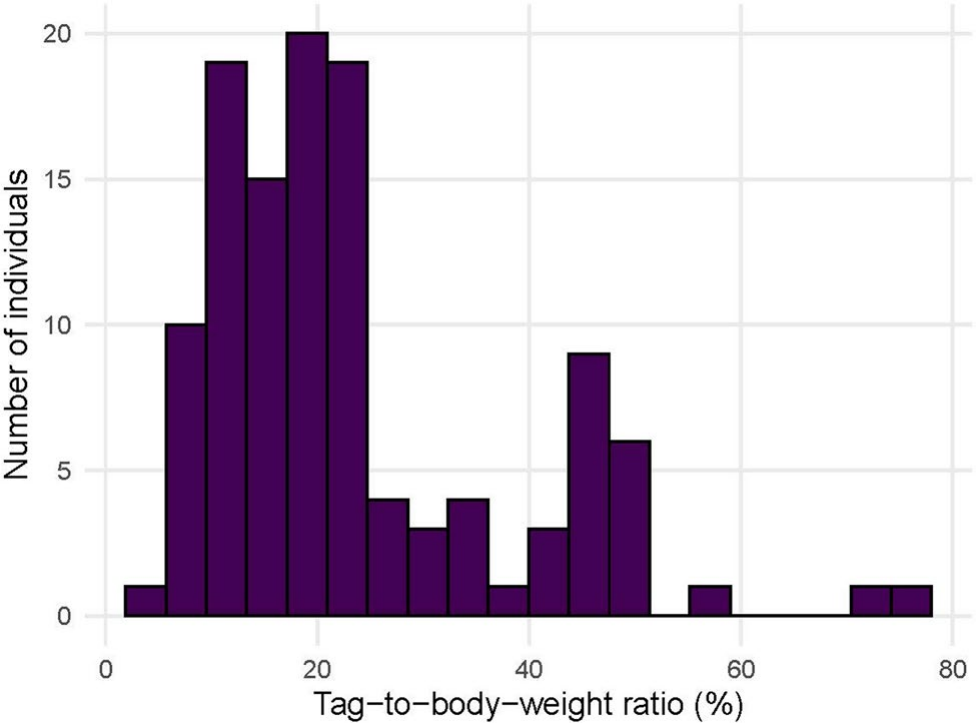
The bars are showing the distribution of tag-to-body-weight ratios across all individuals included in the analysis

A total of 117 butterflies of 18 species were assessed for initial flight performance. The observed tag-to-body-weight ratio ranged from 5.6% to 77.8% (mean = 23.4 ± 14.1%), illustrating the variability in tag burden among individuals (Fig. 3). Overall, 45 individuals showed impaired initial flight (category 1), while initial flight was strong (category 2) in 72 individuals.

**Fig. 4:**
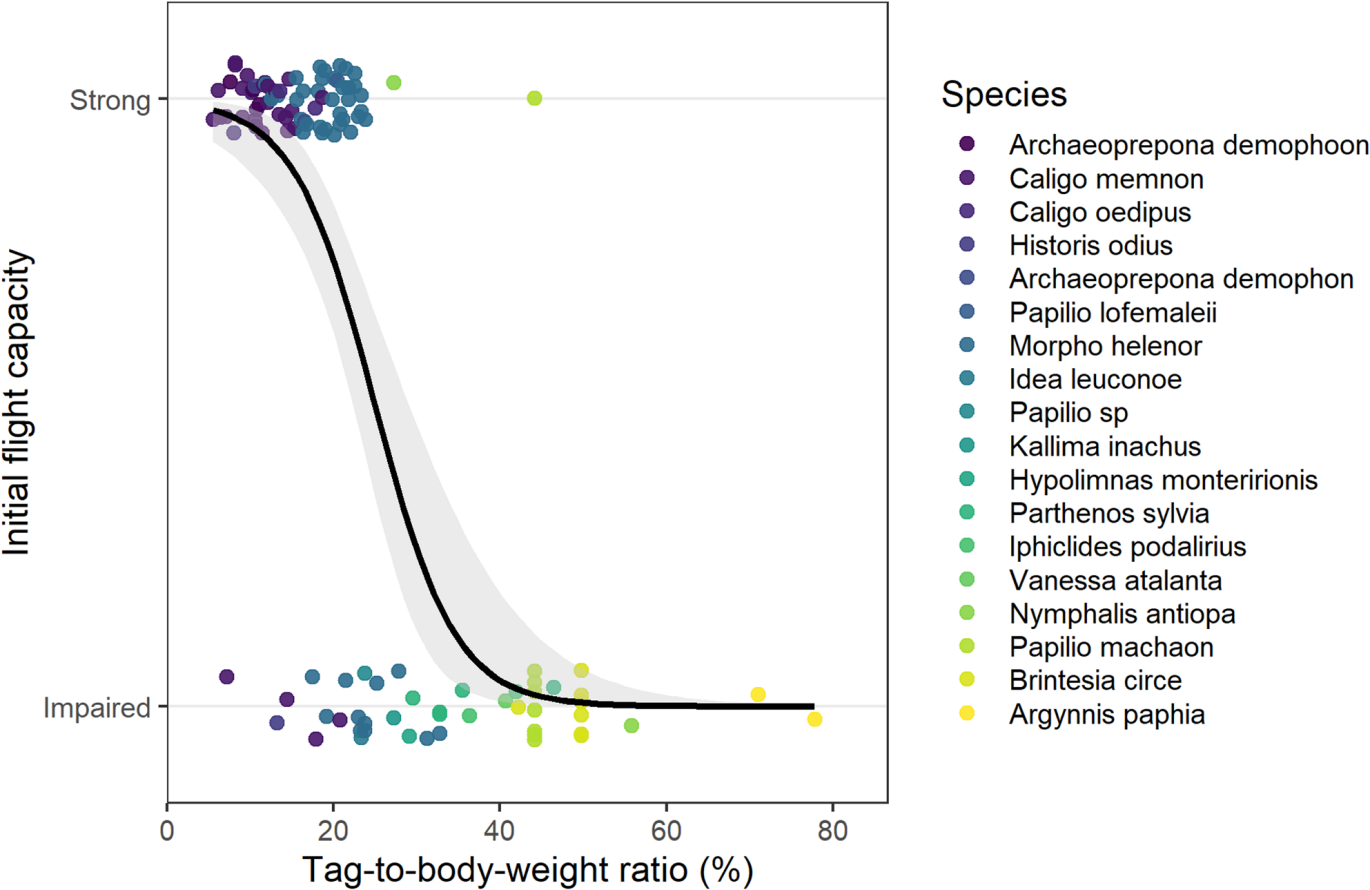
Initial flight capacity (0 = impaired, 1 = strong) of individual butterflies in relation to tag-to-body-weight ratio. Colours denote species. The solid black line shows the fitted logistic regression with 95% confidence interval

The distribution of the raw data across species and the global logistic regression fit are shown in Fig. 4, illustrating the overlap among species and the continuous decrease in flight capacity with increasing tag-to-body-weight ratio.

The GLMM revealed a significant relationship between higher tag loads and reduced likelihood of strong flight (β = -0.213, SE = 0.051, z = -4.15, *p* < 0.001). The odds of displaying strong flight decreased by approximately 19% with each 1% increase in tag-to-body-weight ratio (OR = 0.81, 95% CI: 0.73–0.89).

**Fig. 5:**
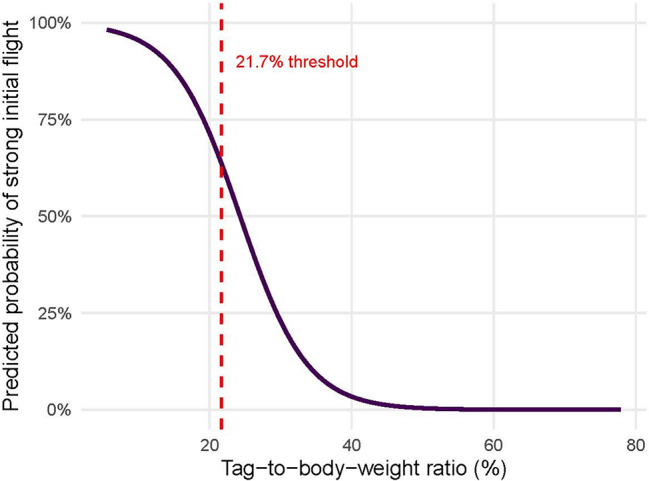
Predicted probability of strong initial flight in relation to tag-to-body-weight ratio. The solid line represents the fixed-effect prediction from the binomial GLMM (logit link) without random effects. The red dashed line indicates the estimated optimal cut-off value of 21.7%, derived from the Youden index of the model’s ROC curve

The model showed excellent discriminatory power, with a receiver operating characteristic (ROC) curve yielding an AUC of 0.91 (Fig. S1). The optimal classification threshold, identified using the Youden index, corresponded to a predicted probability of strong initial flight of 63.3% and translated to a tag-to-body-weight ratio of 21.7% (Fig. 5). Individuals carrying tags above this threshold were substantially less likely to exhibit strong initial flight.

An alternative model including a random slope for the tag-to-body-weight ratio and an observation-level random effect (OLRE) did not improve model fit (ΔAIC = + 4.7), and showed convergence warnings.

### Extended flight performance

**Fig. 6:**
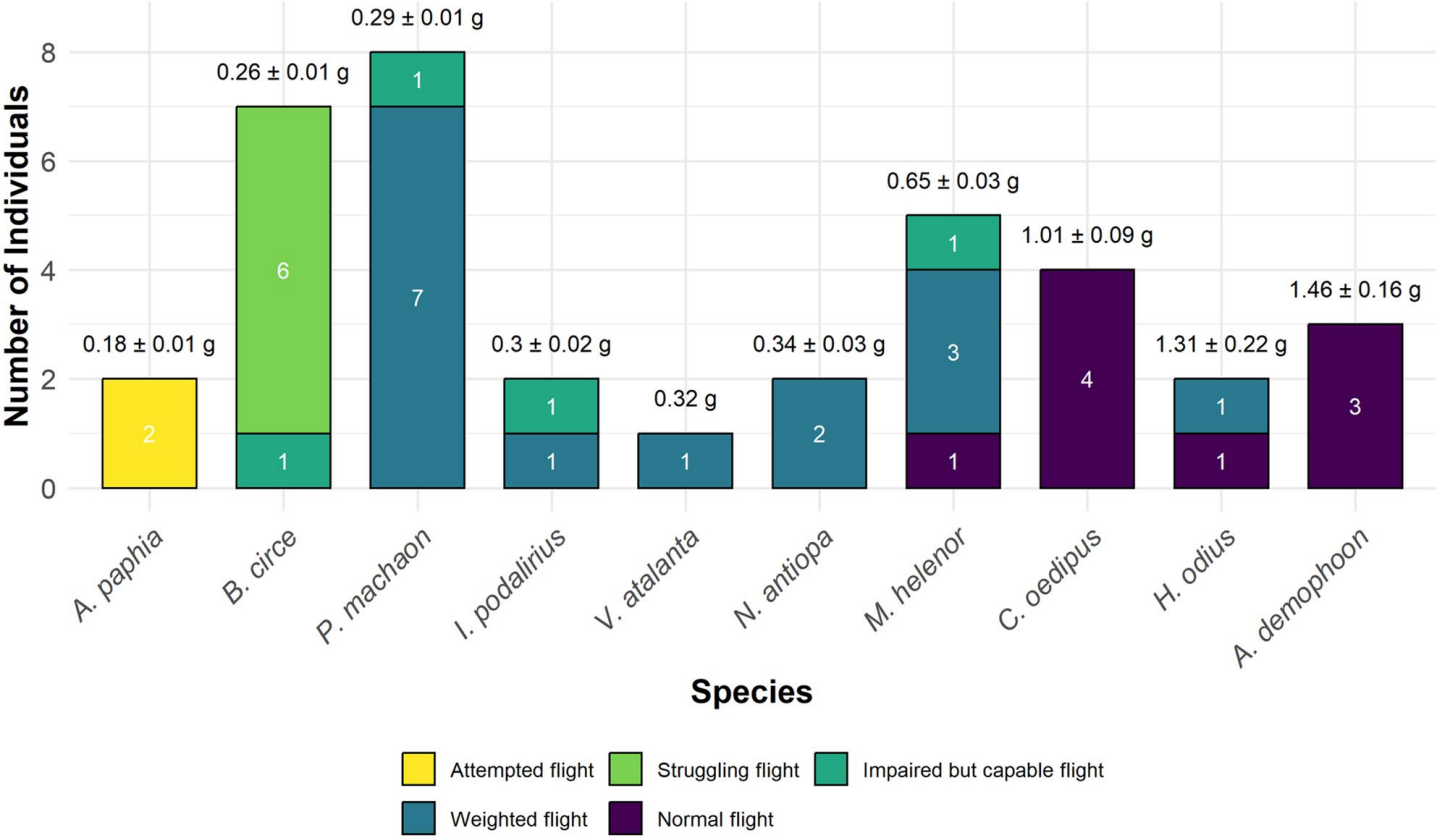
Flight performance of 36 butterflies from 10 species with extended-flight measurements. Bars are stacked by flight performance category: Attempted flight, Struggling flight, Impaired but capable flight, Weighted flight, and Normal flight. Numbers inside each segment indicate sample size. Above each bar, mean body weight ± SE is shown (for *V. atalanta*, single individual weight of 0.32 g)

A total of 36 butterflies from 10 different species were tracked in the field to evaluate their extended flight performance. The individuals exhibited a broad range of flight capacities: 15 butterflies were categorized as weighted flight (category 4), three as impaired but capable flight (category 3), and eight showed severely impaired flight (categories 1 and 2). Normal flight (category 5) was observed in nine individuals (Fig. 6). ‘Normal flight’ was observed only in butterflies with a tag-to-body-weight ratio of ≤ 20%. E.g. *Argynnis paphia*, with a ratio of up to 77.8%, was unable to sustain flight. For 24 out of 36 butterflies, total flight distance was recorded. The mean flight distance with tags attached was 950 m (SD = 1968 m; range: 20–9378 m) within an average observation time of 2.9 days (SD = 1.6 days; range: 1–6 days). No flight distance was recorded for those individuals, which were unable to sustain flight after release, when the tag detached shortly after tagging, or when the signal was lost within a few minutes after release.

The CLMM revealed a significant negative effect of tag-to-body-weight ratio on extended flight capacity (Estimate = -0.22 ± 0.06 SE, z = -3.99, *p* < 0.001), indicating that individuals with higher relative tag loads were more likely to show lower flight performance.

### Tag attachment sites

**Fig. 7:**
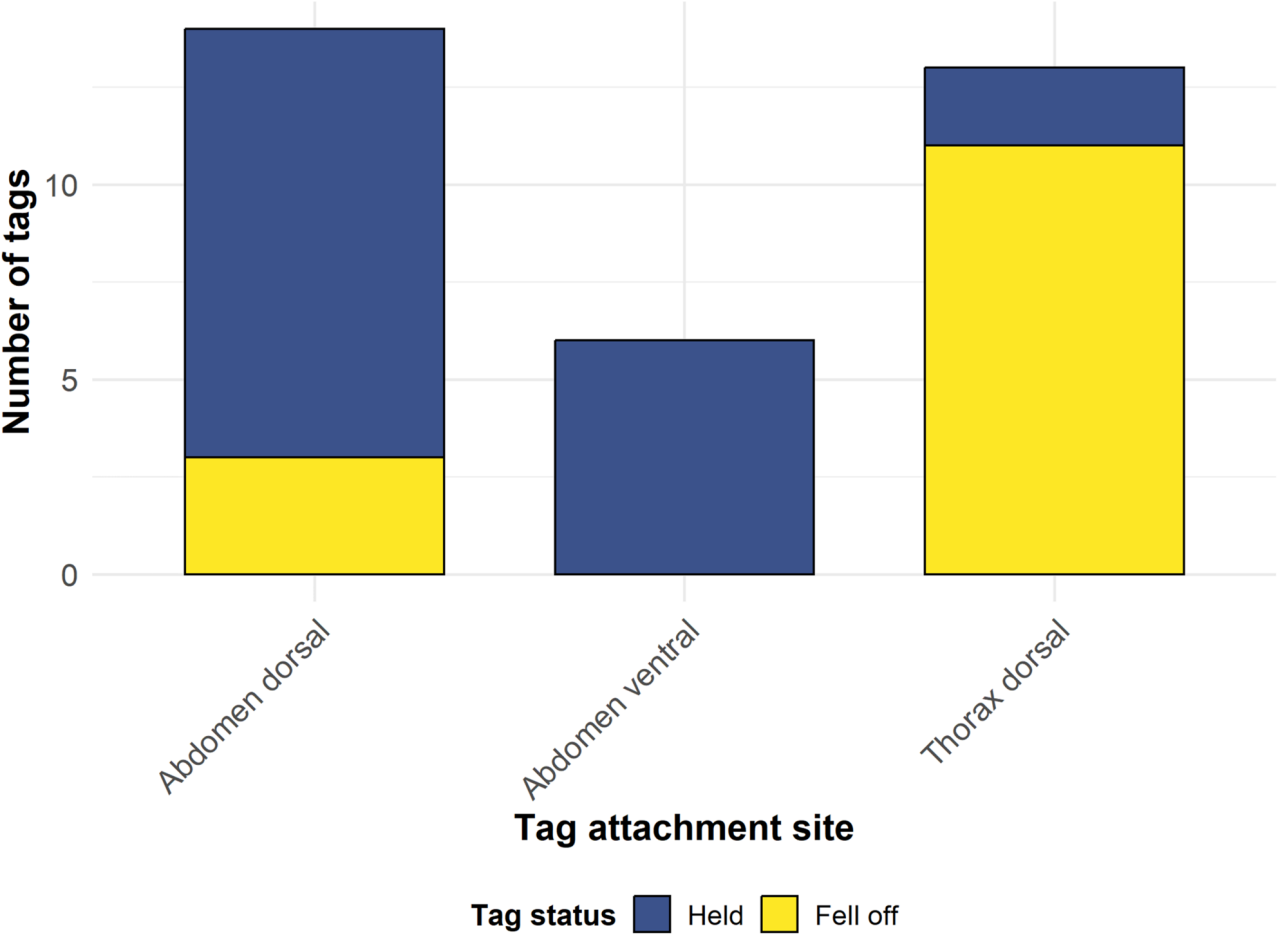
Tag detachment rate as a function of attachment site. Blue bars represent tags that remained attached (held), while yellow bars indicate cases where the tag detached (fell off). Tag detachment was significantly associated with tag placement position (Fisher’s Exact Test, *p* < 0.001)

Based on a sample of 33 butterflies, the tag attachment site significantly influenced tag retention (Fisher’s Exact Test, *p* = 0.00017, two-sided). We revealed a significant association between tag attachment site and detachment rate, with tags adhering more securely to the abdomen than to the thorax (Fig. 7).

## Discussion

### Flight evaluation

Our results consistently show that butterfly flight performance decreases with increasing tag-to-body-weight ratio. The probability of normal flight declined once tag loads exceeded about 20% of body weight. However, these results do neither imply that tag-to-body-weight ratios below 20% have no effect on long-term flight performance, nor that single individuals with a higher tag-to-body-weight ratio are unable to cover considerable flight distances. Flight capacity varied considerably among individuals and between species, even under similar tag loads. In the field, flight distances of over 9 km in *P. machaon* and nearly 4 km in *I. podalirius* were recorded, despite tag weights accounting for approximately 40% of their body weight. While these observations suggest that some individuals may compensate for the added load during extended flight, initial flight performance in these cases was still visibly impaired. Such cases should therefore be regarded as exceptions rather than representative outcomes.

Based on these results we recommend a general threshold of approximately 20% tag-to-body-weight ratio for telemetry studies on butterflies, as this value closely reflects the model-derived cut-off of 21.7%, and offers a practical guideline to minimize the risk of impaired flight performance. With 0.13 g battery-powered tags, radiotelemetry studies can therefore only be suggested for butterflies of more than 0.65 g body weight. Even for larger species, tags are expected to increase energy expenditure to some extent, and such effects may accumulate over time. This means that even if tagged individuals show strong flight within the first days, the tags could potentially affect flight capacity at longer time scales. However, further research is needed to quantify potential long-term effects.

The weight limitations hardly allow telemetry studies of many migratory species such as *Danaus plexippus*, *Vanessa atalanta* and *V. cardui*, as well as many *Pieris* and *Colias* species with 0.13 g light transmitters. However, the 0.06 g light BlūMorpho tags developed in 2023 [36] could be an important step to conduct radiotelemetry studies for some of these species.

Previous research indicated that different load limits apply to various animal groups. Bodey et al., for instance, propose a strict limit of under 1% of body weight for vertebrates, since tag loads between 1% and 5% can negatively impact survival, reproduction, and brood care [37]. Nevertheless, a commonly cited threshold of 5% remains in practice [24]. Since insects, particularly those that provide brood care, such as Hymenoptera, carry heavier loads compared to birds [24, 38, 39], the same thresholds do probably not apply. However, no standardized guidelines for insect telemetry exist, and systematic studies on their carrying capacities are lacking. Also, within insects, threshold values are probably most meaningful when applied within a specific taxonomic group and we emphasize that our results apply specifically to butterflies, and should not be directly extrapolated to other invertebrates, for example moths, which differ in wingbeat frequency and flight mechanics. The study of Boiteau and Colpitts [27] on the Colorado potato beetle (*Leptinotarsa decemlineata*) is frequently cited as the basis for an assumed feasible tag-to-body weight ratio threshold of 23–33% in beetles (e.g [40–42]). However, this is the maximum load that is permissible before the species’ ability to fly is completely lost [18, 27]. Batsleer et al. report that for Hymenoptera [18], especially *Apis mellifera*, the criterion that the additional weight of a device should not exceed an average or maximum nectar or pollen load is often adopted (e.g [43, 44]). In contrast to insects that provide brood care, butterflies are capital breeders, meaning their carried load is predominantly determined by nutrient accumulation during the larval stage rather than by externally gathered resources [45]. Although females often weigh more than males because they carry eggs, tag loads of up to 100% of their body weight – such as those used in bumblebee studies [12] – seem highly impractical for butterflies. However, in some pierid species, males carry their typically larger mates during flight, resulting in reduced flight performance [46] and likely increased predation risk. Almbro & Kullberg documented that mate carrying produces negative take-off angles and hampers the male’s ability to gain altitude [46]. In butterflies, carrying additional load is generally unusual and limited to specific situations, such as females carrying developing eggs or being transported during mating. While some butterflies may also temporarily carry foreign material, such as water droplets or pollen adhering to their body, these instances are rare and typically short-lived. Load tolerance may not only depend on body weight but also on species-specific differences in flight behavior, wing morphology, and aerodynamic performance. For example, Le Roy et al. demonstrated that species within the genus *Morpho* exhibit distinct flight strategies and wing morphologies depending on their vertical forest habitat [47, 48]. Canopy-dwelling species showed increased glide efficiency and specialized morphological traits. Such differences in flight mechanics and morphology may influence the aerodynamic impact of added weight and thus help explain why species differ in their tolerance to external loads.

In addition to interspecific variation, intraspecific differences in wing loading may also be important. Since volume i.e. body mass increases with the cube of size, while surface area i.e. wing span increases only with the square, larger individuals of the same shape experience relatively higher wing loading and may therefore be disproportionately affected by added tag weight. Previous work of Batsleer et al. demonstrated that tag effects were more strongly associated with wing loading than with absolute body weight [18]. Such effects can bias results if body size correlates with life-history traits. In our analysis of *Caligo* and *Morpho*, wing loading did not outperform body weight ratio (Table S1), but it remains an important parameter to consider when evaluating within-species variation.

### Tag attachment sites

We observed better tag attachment when radio transmitters were attached to the abdomen instead of the thorax of butterflies. The ductile abdomen may provide a larger contact surface for the adhesive compared with the relatively stiff thorax, resulting in smaller tag loss rates. In addition, flight muscles might cause subtle movements of the thorax with every wing beat, which may eventually cause the hardened adhesive to loosen and, consequently, the radio tag to fall off more quickly. Therefore, we believe, tag attachment to the abdomen is advantageous from a technical perspective.

In contrast, a thoracic attachment could be biomechanically advantageous, as the tag is then closer to the flight muscles, where the center of gravity of the body may also be located. At the same time, thoracic attachment may subtly interfere with wing movement or muscle efficiency, which could compromise flight performance. The exact position of the body’s center of gravity seems to depend on certain adaptations [49, 50]. Srygley & Dudley investigated how the position of the body’s center of gravity affects maneuverability and flight speed in neotropical butterflies [50]. Palatable butterflies were found to fly faster and evade predator attacks more effectively due to their center of gravity being positioned closer to the wing base [50]. They also tend to have a larger thorax and a smaller abdomen. In contrast, unpalatable butterflies – those that are chemically defended – have a more posterior center of gravity [50], which may influence their susceptibility to predation. It is therefore possible that not the tag weight alone but also the shift in the center of gravity could affect predation risk of radio tagged butterflies, when the transmitter is attached to the abdomen. In addition, attachment to the abdomen could interfere with oviposition or the mating process, especially since the antenna extends beyond the abdomen. It is unlikely that a butterfly perceives the tag’s and antenna’s dimensions and adjusts its movements accordingly. The antenna could also increase the chance that butterflies become entangled in dense vegetation or spider webs. A similar finding was reported by Dubois and Vignon [42]. Here, beetles of *Osmoderma eremita* equipped with tags occasionally became entangled in thin branches and leaves due to the tags.

### Limitations

This study examined initial flight ability but did not assess the overall behavior of butterflies after tagging. Studies on birds have shown that individuals with attached tags exhibit behavioral changes in the initial phase after handling, suggesting that they first need to acclimate to the tag. A study on Ruby-throated Hummingbirds (*Archilochus colubris*) found that flight time and, consequently, modeled flight distance decreased after tagging [51]. In other bird studies, tagged individuals initially exhibited altered behavior compared to the control group, such as spending more time on preening. However, over time, behavioral differences disappeared [52, 53].

Similarly, Monarch butterflies (*D. plexippus*) spent less time in the air during a 20-minute observation period immediately after being equipped with sham tags [21]. In our study, two individuals of *M. helenor*, were observed attempting to remove the tag with their legs by tilting the abdomen forward in flight and pressing their legs against the tag. This indicates that the tag is perceived as a foreign body and can trigger a stress reaction. A crucial open question remains whether other species also show this behavior, how long it lasts and if the tags represent an initial or permanent disturbance.

In addition to these behavioral responses, practical aspects that are crucial when handling butterflies during field work must also be taken into account. For the use of radio telemetry in field studies on butterfly movement ecology, we emphasize the need for continuous tracking. Only when the signal is tracked throughout the day a sudden and unnoticed loss of the signal can be avoided. The continuous tracking requires sufficient personnel or automated radio telemetry systems [54].

Tracking is generally feasible in open landscapes, sparse woodland, and even in forests, though progress in dense vegetation is slower and visibility is often limited. Urban infrastructure can also obstruct the signal and restrict access. At least two observers are recommended to reliably follow moving individuals, to alternate roles, and maintain attention over longer periods.

With some experience, tagged butterflies can often be located with high precision. Visual observations of behavior (e.g. resting, feeding, mating) are often possible, though binoculars are often needed, especially when butterflies are resting in the canopy.

A combination of radio telemetry with capture-mark-recapture studies could help to investigate butterfly mobility and behavior more comprehensively. Comparing results from both methods could reveal whether mobility and behavioral patterns inferred from capture-mark-recapture studies align with those obtained through radio telemetry, providing a more robust understanding of butterfly movement ecology. This approach has already been applied to beetles, where comparable results were obtained regarding dispersal patterns [55, 56].

## Conclusions

Our study provides valuable methodological insights for butterfly telemetry. We demonstrate that the tag-to-body-weight ratio has a significant impact on butterfly flight performance and identified a threshold of approximately 20% of body weight, beyond which flight impairment becomes evident. While some individuals were still able to fly with a higher tag-to-body-weight ratio, their initial flight ability after tag attachment was visibly reduced. This finding is crucial for future butterfly telemetry studies, as exceeding this threshold may alter natural flight behavior and increase energy expenditure of the butterflies.

The attachment site plays a key role in tag retention. While abdominal attachment ensured better adhesion, thoracic attachment could be biomechanically advantageous due to its proximity to the flight muscles. These trade-offs and species-specific characteristics should be carefully considered when choosing the appropriate tag attachment site in future butterfly telemetry studies.

Our results confirm the successful application of radio telemetry for studying butterfly movement and behavior, but they also highlight methodological challenges, including optimal tag placement and possible behavioral changes in butterflies after tagging. We believe that the results of our study provide an important step for making radio telemetry a key method for studying butterfly movement ecology. With the battery-powered radio tags (0.13 g), we propose the use of radio telemetry for selected butterfly species with a mean body weight of ≥ 0.65 g. The next generation of lighter tags based on solar energy will expand the range of species in the future. We encourage future studies to examine the medium-term effects of tagging on butterfly survival, predation risk, and reproduction. We eagerly await the development of additional lightweight tags to make radio telemetry applicable to a larger number of butterflies and other insects.

## Supplementary Information

Below is the link to the electronic supplementary material.

Supplementary Material 1

Supplementary Material 2

Supplementary Material 3

## Data Availability

The anonymized dataset and R code used for the logistic regression analysis are provided as supplementary material and will be archived publicly on Zenodo upon acceptance of the manuscript. Other statistical procedures were minor and are described in the manuscript.

## Abbreviations

AUC: Area Under the Curve
CMR: Capture–Mark–Recapture
DFG: Deutsche Forschungsgemeinschaft
GLMM: Generalized Linear Mixed–Effects Model
GPS: Global Positioning System
OLRE: Observation–Level Random Effect
QGIS: Quantum Geographic Information System
R: R programming language
ROC: Receiver Operating Characteristic
SD: Standard Deviation
SE: Standard Error
VHF: Very High Frequency
