# Supplementary material for: Radio telemetry of butterflies: practical insights and critical tag-weight thresholds
Source: Mov Ecol. 2025 Dec 9;13:86. doi: 10.1186/s40462-025-00615-9 (PMC12690926; doi:10.1186/s40462-025-00615-9)
Supplement: Supplementary file 2 — Supplementary Material 2 [file 40462_2025_615_MOESM2_ESM.pdf]

Radio telemetry of butterflies: practical insights and critical tag-weight thresholds

Supplementary Figures and Tables

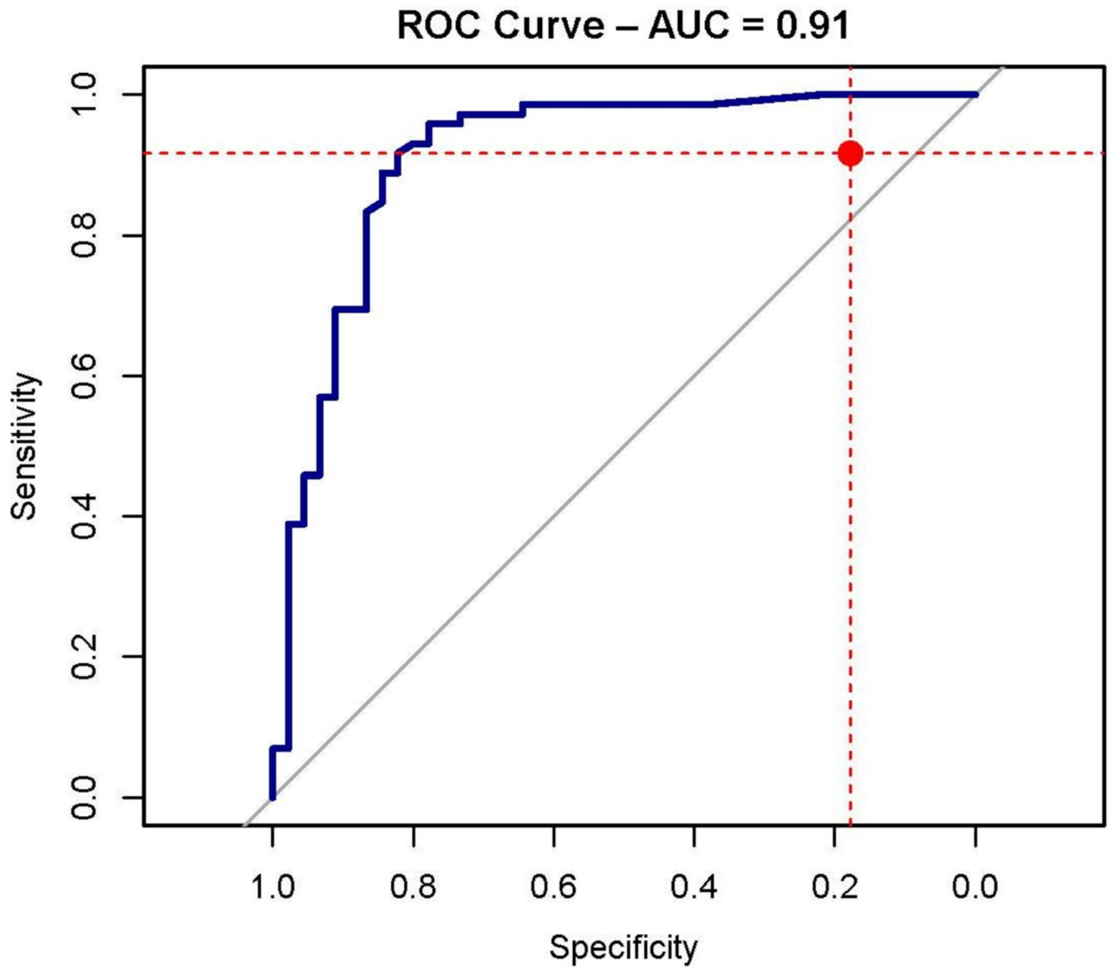

Figure S1: Receiver operating characteristic (ROC) curve illustrating the performance of the binomial GLMM in predicting initial flight capacity. The area under the curve (AUC) was 0.91, indicating high discriminatory power. The red point marks the optimal classification threshold as determined by the Youden index, with dashed lines indicating the corresponding sensitivity and specificity.

Table S1: Logistic regression results comparing the predictive performance of tag-to-body-weight ratio and tag-to-wing-loading ratio for initial flight capacity in *Caligo* and *Morpho* butterflies (Biosphere Potsdam). Wing loading was approximated using forewing length squared. Only the body-weight ratio was significant and consistently provided a better model fit (lower AIC).

Genus	Model	Estimate	SE	z	p	AIC
Caligo	Tag-to-body-weight ratio	−1.09	0.62	−1.78	0.076	23.8
Caligo	Tag-to-wing-loading ratio	−0.74	0.58	−1.28	0.200	25.8
Morpho	Tag-to-body-weight ratio	−1.94	0.79	−2.47	0.014	35.5
Morpho	Tag-to-wing-loading ratio	−0.69	0.44	−1.56	0.119	46.1
